# 

**Published:** 1951-07

**Authors:** 


### Local Medical Notes

**The XL Long Fox Memorial Lecture** will be delivered in the University on 16th October, 1951, by Professor C. F. Powell, F.R.S., Sc.D., Melville Wills Professor of Physics in the University of Bristol. Subject: **"Physical Principles of Radioactivity and the Release of Atomic Energy".**

### Appointments

N. F. W. Brueton.—Deputy Director, South-Western Regional Blood Transfusion Service.

G. H. Darke, F.R.C.S.—Consultant Surgeon, South Somerset.

C. C. Jeffery, F.R.C.S.—Consultant Orthopaedic Surgeon, Exeter.

F. R. Hurford, F.R.C.S.—Consultant Surgeon, Lichfield, Sutton, and Tamworth Hospitals.

T. C. H. Davies, F.R.C.S.—Consultant Orthopaedic Surgeon, Newport.

P. Jardine, F.R.C.S., D.O.M.S.—Consultant Ophthalmic Surgeon, Southmead Hospital, Bristol.

John Winter, M.B., M.Rad., D.M.R.D.—Consultant Radiologist, South Somerset Clinical Area.

J. H. Bergin, M.B., B.S., D.M.R.D.—ConsultantRadiologist, Swindon Area.

### University of Bristol

#### Prizes

*Foyle.*—Miss M. E. Moore.

*Edward Fawcett Memorial.*—Miss M. E. Moore.

*Francis Dudley.*—Mr. H. Savery.

*Richard Clarke.*—Miss J. K. Lloyd.

### Degrees and Diplomas

**University of Bristol.**—D.M.R.D.: Sheila M. Cameron, A. G. M. Davies.

**Royal College of Physicians.**—F.R.C.P.: Dr. H. D. Davies, Dr. J. E. G. Pearson.

